# 

**Published:** 1903-05

**Authors:** 


### East Texas Medico-Chirurgical Society.

The sixth semi-annual session of the East Texas Medico-Chirurgical Society will be held Thursday and Friday, May 28 and 29, 1903, at City Hall, Palestine, Texas.

*Officers.*—Dr. A. L. Hathcock, President, Palestine, Texas; Dr. E. B. Parsons, First Vice-President, Palestine, Texas; Dr. Jno. H. Joyce, Second Vice-President, Buffalo, Texas; Dr. J. M. Colly, Treasurer, Palestine, Texas; Dr. E. E. Guinn, Secretary, Jacksonville, Texas.

